# Gut Microbiota Accelerate the Insecticidal Activity of Plastid-Expressed Bacillus thuringiensis Cry3Bb to a Leaf Beetle, *Plagiodera versicolora*

**DOI:** 10.1128/spectrum.05049-22

**Published:** 2023-03-28

**Authors:** Xiaoyu Lei, Fengjuan Zhang, Jiang Zhang

**Affiliations:** a State Key Laboratory of Biocatalysis and Enzyme Engineering, Hubei Hongshan Laboratory, School of Life Sciences, Hubei University, Wuhan, China; b Agricultural Genomics Institute at Shenzhen, Chinese Academy of Agricultural Sciences, Shenzhen, China; China Agricultural University

**Keywords:** plastid transformation, *Bacillus thuringiensis* crystal protein, gut microbiota, poplar, leaf beetle

## Abstract

Bacillus thuringiensis is widely used as a biopesticide, and its crystal protein expressed in transgenic crops has been successfully used for the management of insect pests. However, whether the midgut microbiota contribute to the *Bt* insecticidal mechanism remains controversial. We previously demonstrated that transplastomic poplar plants expressing *Bt* Cry3Bb are highly lethal to willow leaf beetle (*Plagiodera versicolora*), one of the major pests causing severe damage to *Salicaceae* plants such as willows and poplars. Here, we demonstrate that feeding poplar leaves expressing Cry3Bb to nonaxenic *P. versicolora* larvae leads to significantly accelerated mortality, and overgrowth and dysbiosis of the gut microbiota, compared with axenic larvae. Corroborating work done with Lepidopteran insects, plastid-expressed Cry3Bb can cause the lysis of the beetle's intestinal cells, lead to the entry of intestinal bacteria into the body cavity, and thus cause the dynamic changes in the flora of the midgut and blood cavity in *P. versicolora*. Reintroduction of Pseudomonas putida, a gut bacterium of *P. versicolora*, into axenic *P. versicolora* larvae further enhances mortality upon feeding on Cry3Bb-expressing poplar. Our results indicate the important contribution of host gut microbiota in promoting the B. thuringiensis crystal protein insecticidal activity and provide new insights into the mechanism of pest control by *Bt*-transplastomic approaches.

**IMPORTANCE** The contribution of gut microbiota to Bacillus thuringiensis Cry3Bb insecticidal activity in a leaf beetle was demonstrated using transplastomic poplar plants, providing a potential new approach to improve the efficiency of plastid transformation technology for pest control by expression of *Bt* toxins.

## INTRODUCTION

Bacillus thuringiensis (*Bt*) is a Gram-positive spore-forming pathogen that targets insects and other invertebrates. It has been successfully applied for pest control for almost a century by either being directly used as a biopesticide or incorporated into genetically modified crops with its toxin genes (1, 2). Toxins produced by *Bt* include insecticidal Crystal (Cry) proteins, vegetative insecticidal protein (Vip) toxins, and cytotoxin (Cyt) proteins (3 to 5). The classical pore-forming model is a widely accepted mode of action of Cry protein, including crystal protein activation by midgut proteases, binding of a specific receptor of the midgut epithelium, and pore formation, which leads to cell lysis and eventual death of the insects (6 to 9).

Insects and their microbiota frequently cohabit harmoniously, and this partnership is generally advantageous to each other (10). The gut microbiota have been recognized as a virtual “organ” that can be integrated into the biological system of their hosts (11 to 13). Coexistence between the insects and their microbiota is mostly harmonious, and in most cases is beneficial to the insects (14). For instance, the gut microbiota have a variety of favorable functions for their host insects, such as facilitation of nutrient assimilation (15, 16), acquirement of resistance against pathogens (17, 18), and detoxification of xenobiotics or poisonous dietary components (19, 20). However, in some cases, the gut microbiota may also be associated with detrimental interactions with the insects. For example, the pathogenic fungus Beauveria bassiana interacts with the gut microbiota to accelerate mosquito death (10), and gut bacteria accelerate the B. bassiana infection process in a bark beetle (21). Moreover, it was shown that the gut microbiota could play a synergistic role in double-stranded RNA-induced mortality of *Plagiodera versicolora*, a leaf beetle of *Salicaceae* plants such as willows and poplars (22).

Although the action mode of *Bt* Cry proteins is clearly presented, whether gut microbiota play a role in the mode action of *Bt* Cry proteins is still an ongoing debate (23 to 32). Although the vast majority of the studies that supported the requirement for gut bacteria for *Bt* pathogenicity used commercially available *Bt* strains (23, 25, 33), some studies on *Manduca sexta* (26) and *Plutella xylostella* (30) demonstrated that the gut bacteria are not required for the decreased insecticidal efficacy of *Bt*. It was proposed that the antibiotics utilized in Broderick's experiment may have an impact on the pathogenicity of *Bt* (26, 30), and live bacterial spores would significantly increase toxin-induced mortality (30). However, these views were extensively refuted by (i) the use of heat-killed E. coli rather than *Bt* to deliver the toxin, (ii) reintroduction of gut bacteria leading to restoration of killing (23), (iii) use of cell-free Cry toxin (25), and (iv) nonantibiotic-treated larvae with cell-free toxin and direct injection of a member of the gut microbiota into the insect's hemocoel (27).

Previously, we have introduced *Bt-Cry3Bb* into the plastid genome of poplar by biolistic bombardment, and the transplastomic poplar is highly lethal to the leaf beetle *P. versicolora* (34). Here, by using aseptic poplar plants and axenic *P. versicolora* larvae (from surface-sterilized eggs), we have explored whether the gut microbiota is involved in the killing process of feeding *P. versicolora* larvae with plastid-expressed Cry3Bb resulting in intestinal perforation. We found that the mortality of axenic larvae fed on the leaves of *Cry3Bb*-expressing poplar is lower than that of nonaxenic larvae. Our results indicate that the gut microbiota contributes substantially to the mortality of *P. versicolora* induced by oral delivery of *Bt* toxin expressed in the plastids of poplar.

## RESULTS

### Gut microbiota accelerate plastid-expressed Cry3Bb-induced mortality of *P. versicolora* larvae.

**FIG 1:**
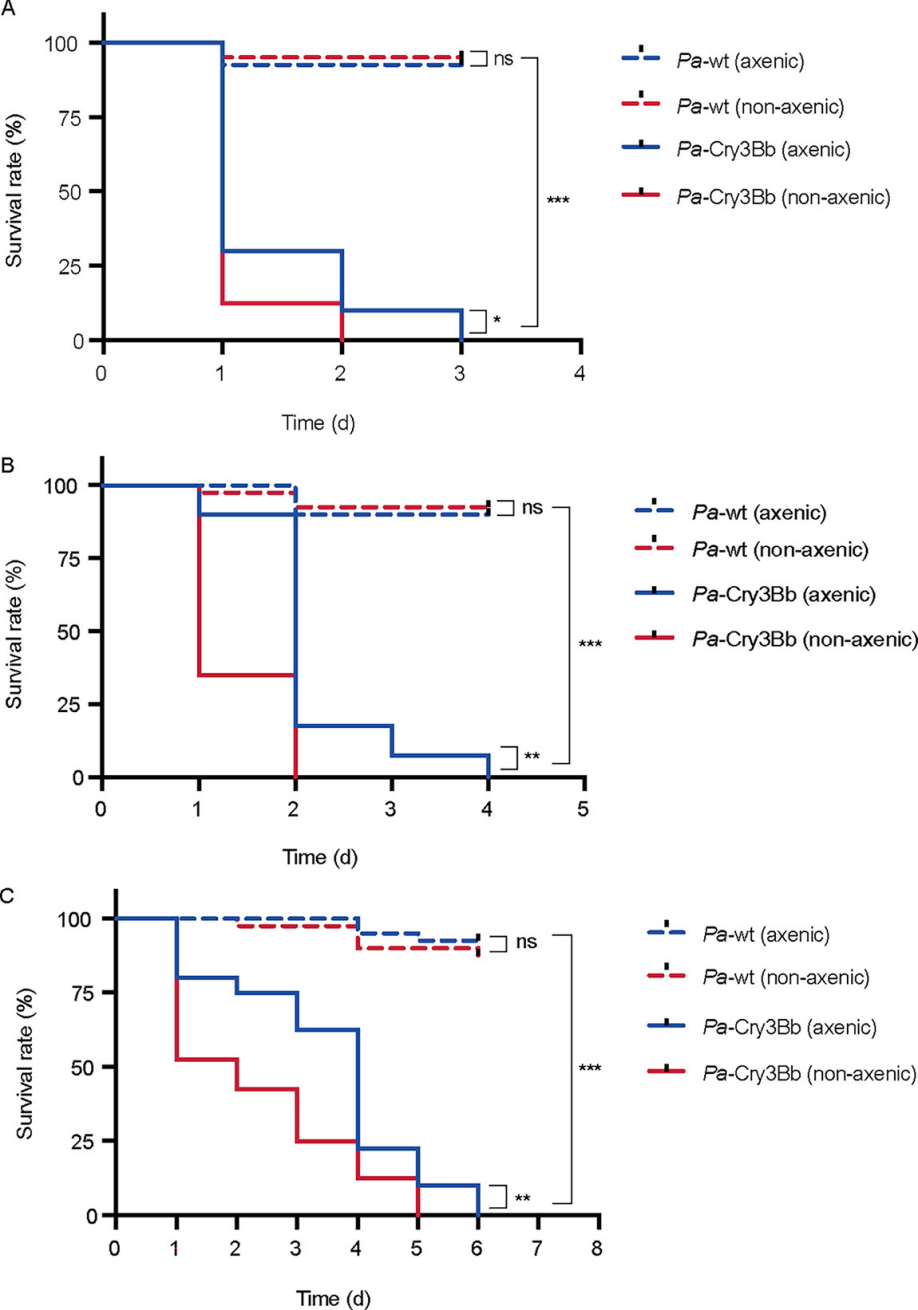
Kaplan-Meier survival curves of second (A), third (B), and fourth (C) instar nonaxenic and axenic *P. versicolora* larvae fed on wild-type poplar (*Pa*-wt) and *Pa*-Cry3Bb (*n *= 40). Blue dotted line: axenic larvae fed on wild-type poplar; red dotted line: nonaxenic larvae fed on wild-type poplar; blue solid line: axenic larvae fed on *Pa*-Cry3Bb; red solid line: nonaxenic larvae fed on *Pa*-Cry3Bb. The log-rank test was used to assess the significance of differences between two survival curves. *, *P < *0.05; **, *P < *0.01; ***, *P < *0.001; NS, not significant.

To evaluate whether the gut microbiota is involved in the insecticidal effects of *P. versicolora* fed on Cry3Bb-expressing poplar plants, axenic larvae were obtained by surface sterilization of eggs (see Materials and Methods). When feeding with aseptic detached leaves of wild-type poplar (*Pa*-wt), no significant differences were revealed in survival of axenic larvae compared with nonaxenic larvae, independent of the larval stage. In contrast, nonaxenic larvae fed with transplastomic poplar line *Pa*-Cry3Bb (34) were killed significantly faster than axenic larvae (Fig. 1A to C). These observations suggest that the gut microbiota could promote plastid-expressed Cry3Bb-induced mortality of *P. versicolora*.

### Feeding of *Pa*-Cry3Bb leads to disruption of intestinal epithelial barrier function.

**FIG 2:**
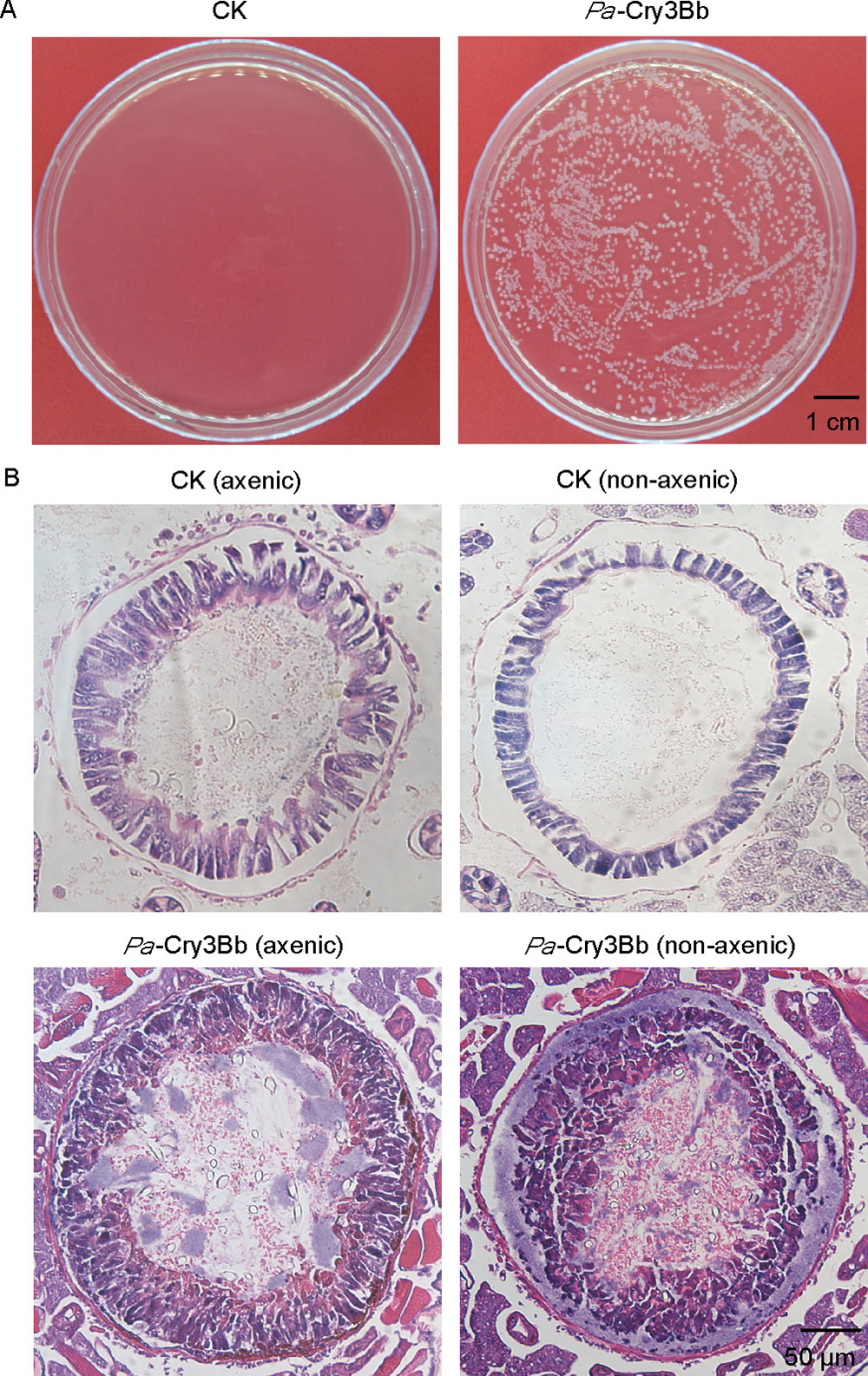
The translocation of gut bacteria to the hemocoel of *P. versicolora* larvae after ingestion of *Pa*-Cry3Bb. (A) The presence of bacteria was determined by plating homogenates of hemolymph fluid obtained from third-instar *P. versicolora* larvae fed with *Pa*-Cry3Bb onto LB agar plates. The larvae fed with *Pa*-wt (CK) that served as the control. (B) The midgut morphology of nonaxenic and axenic *P. versicolora* larvae fed on *Pa*-wt (CK) and *Pa*-Cry3Bb. Midgut cross sections were stained with hematoxylin and eosin. Scale bars: 50 μm.

To explore whether the lysis of the gut epithelium is responsible for the enhanced killing efficiency, we extracted hemolymph from nonaxenic larvae fed with *Pa*-Cry3Bb with a capillary and examined the sample for the presence of bacteria by culturing it on LB medium. As expected, although no bacterial colonies grew from axenic larvae, substantial colonies formed from hemolymph samples obtained from nonaxenic larvae fed with *Pa*-Cry3Bb (Fig. 2A). Moreover, histological analysis revealed that the gut epithelia of both axenic and nonaxenic larvae fed on *Pa*-Cry3Bb were disrupted, whereas they remained intact in the wild-type controls. In addition, the damaged levels of the gut epithelium were more severe in nonaxenic larvae than in axenic larvae (Fig. 2B). These observations provide further evidence that gut bacteria enter the body cavity through Cry3Bb-induced epithelial damage.

### Feeding of *Pa*-Cry3Bb results in alteration of the gut bacteria in *P. versicolora*.

**FIG 3:**
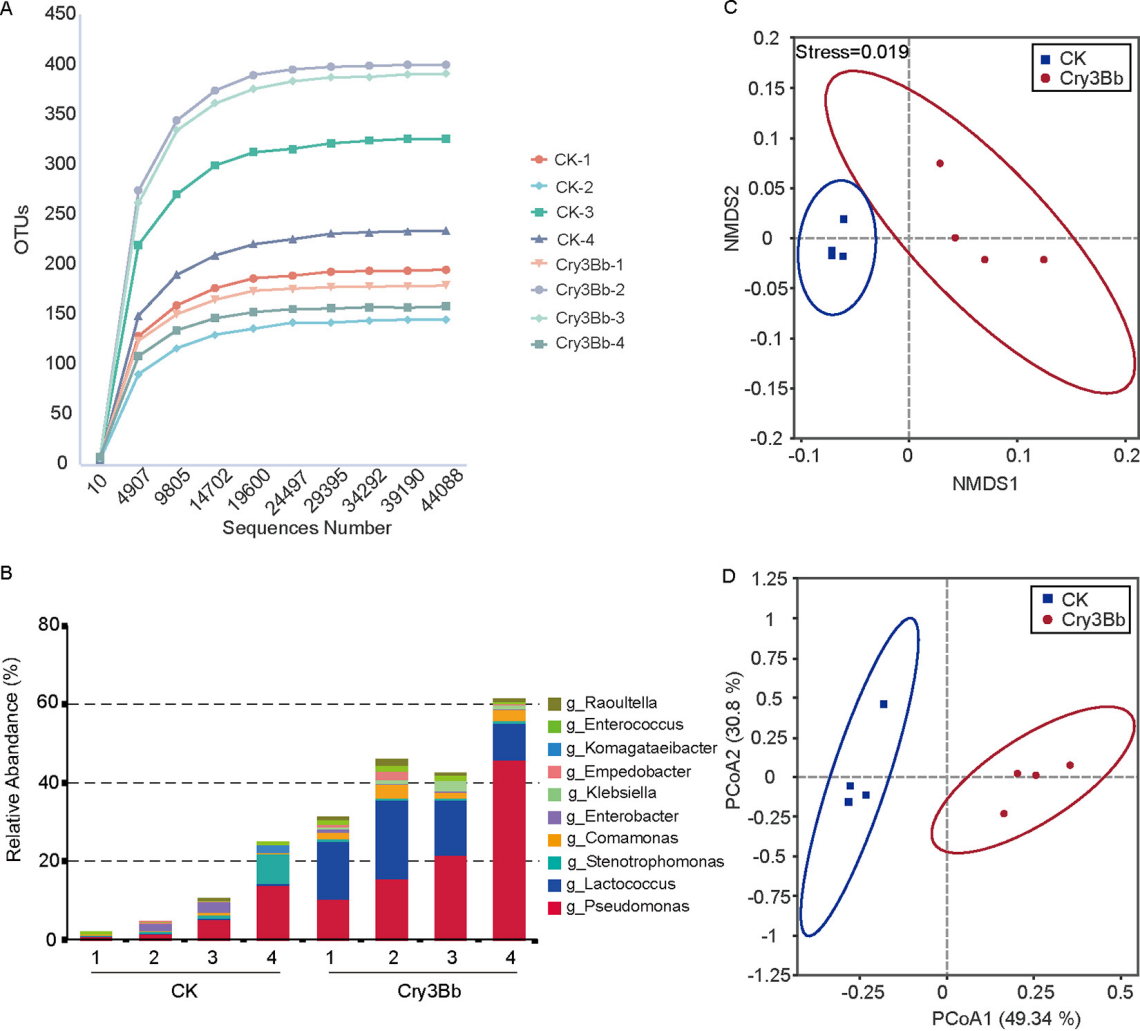
Cry3Bb treatment alters the composition of gut microbiota in *P. versicolora*. (A) Rarefaction curves of 8 gut samples based on Miseq sequencing of bacterial communities. (B) The top10 bar plot of relative abundance of genus between *Pa*-wt (CK) and *Pa*-Cry3Bb (Cry3Bb) treatment gut microbiota in *P. versicolora*. (C) Nonmetric multidimensional scaling (NMDS) diagrams of 8 samples, based on weighted UniFrac. (D) Principal coordinate analysis (PCoA) plots based on the Bray–Curtis metric for bacterial communities. Permutational multivariate analysis of variance indicated that the bacterial community of samples fed with *Pa*-wt (CK) larvae was significantly different from that fed with *Pa*-Cry3Bb (Cry3Bb) larvae (ADONIS, *P < *0.05).

We first analyzed the composition and diversity of the midgut bacteria in the untreated *P. versicolora* larvae feeding on wild-type (*Pa*-wt) and *Pa*-Cry3Bb poplar plants by deep sequencing of the 16S rRNA genes. Alpha diversity was estimated using four measurements: Chao1 index, Observed_otus, Shannon–Weiner index, and Simpson’s index (Table S1 in the supplemental material). In general, no significant differences were found for the α-diversity indices between the gut samples of the *P. versicolora* larvae feeding on wild-type and *Pa*-Cry3Bb poplar plants. Rarefaction curves of the 8 samples almost reached equilibrium, indicating that the natural bacterial diversity was well covered by the sequencing analysis (Fig. 3A). In the *P. versicolora* larvae feeding on wild-type poplar, the midgut bacteria were diverse and mainly dominated by five bacterial genera: Pseudomonas, Enterobacter, Stenotrophomonas, Comamonas, and Lactococcus (Fig. 3B). The proportion of Pseudomonas, *Lactococcus*, and *Comamonas* was significantly increased, while the proportion of Enterobacter and *Stenotrophomonas* was decreased in the *P. versicolora* larvae feeding on *Pa*-Cry3Bb leaves compared to the control larvae (feeding on wild-type poplar). Nonmetric multidimensional scaling (NMDS) ordination based on weighted UniFrac showed that the bacterial communities of the control larvae (feeding with wild-type poplar) and the *Pa*-Cry3Bb-feeding larvae were separately clustered (Fig. 3C; ANOSIM, *P < *0.05). A phylogeny-based Bray–Curtis principal coordinate analysis (PCoA) considering relative abundances of ASVs (Amplicon Sequence Variants) showed a similar result (Fig. 3D; ADONIS, *P < *0.05). These results suggest that feeding *Pa*-Cry3Bb to *P. versicolora* larvae resulted in the dramatic change of the composition and diversity of the midgut microbiota.

### Reintroduction of gut bacteria into axenic *P. versicolora* larvae enhances mortality upon administration of *Pa*-Cry3Bb.

**FIG 4:**
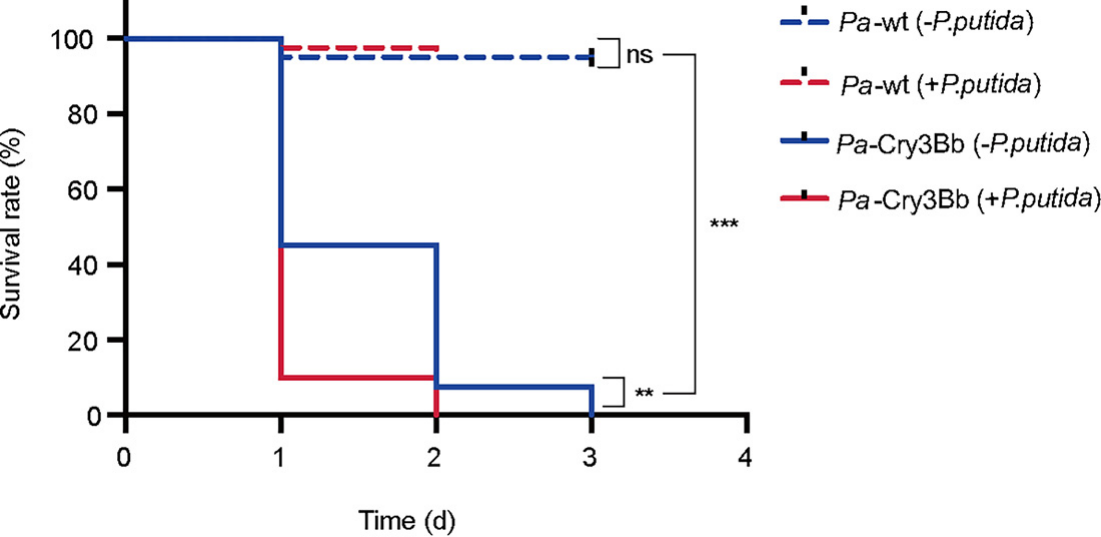
Kaplan–Meier survival curves of axenic *P. versicolora* larvae (*n *= 40) fed on *Pa*-wt and/or *Pa*-Cry3Bb after reintroduction of gut bacteria P. putida. Blue dotted line: axenic larvae not reintroduced with P. putida, and then fed on *Pa*-wt; red dotted line: axenic larvae reintroduced with P. putida, and then fed on *Pa*-wt; blue solid line: axenic larvae not reintroduced with P. putida, and then fed on *Pa*-Cry3Bb; red solid line: nonaxenic larvae fed on *Pa*-Cry3Bb and then fed on *Pa*-Cry3Bb. The log-rank test was used to assess the significance of differences between the two survival curves. **, *P < *0.01; ***, *P < *0.001.

To ultimately confirm the effect of gut bacteria on Cry3Bb-induced mortality of *P. versicolora*, we reintroduced Pseudomonas putida (isolated from the gut of *P. versicolora*) (22) into axenic larvae. The inoculation with bacteria did not have negative effects on the survival of larvae fed on wild-type poplar. However, upon feeding with *Pa*-Cry3Bb, larvae inoculated with bacteria were killed significantly faster than untreated larvae (Fig. 4; log-rank test, *P < *0.05).

## DISCUSSION

Whether the gut microbiota is required for the pathogenesis of *Bt* crystal protein is a long-lasting controversy for the action mechanism of *Bt* crystal protein. The controversy may be due to the experimental manipulation that could lead to different results. First, antibiotic treatment normally was used to render larvae aseptic; however, the presence of antibiotics may reduce the pathogenicity of *Bt* (30). Second, most of the experiments were conducted with live *Bt* strains, which greatly increased toxin-induced mortality (35, 36). This makes it difficult to determine whether the gut bacteria or the live *Bt* strains were responsible for the insect mortality.

In the present work, to circumvent the impacts of the antibiotics treatment and live *Bt* strains, we obtained axenic larvae by surface sterilization of eggs (22) and explored whether the gut microbiota play a key role in the killing process of Cry3Bb expressed in plastids of poplar plants (34). In a sterile environment, the axenic larvae were fed with aseptic leaves of poplar plants to maintain an axenic status. Compared with the antibiotic treatments (17, 32, 37), this method can ensure the complete removal of microbes and avoid the additional influence on larval development upon hatching (28). By feeding leaves of transplastomic poplar plants expressing Cry3Bb (*Pa*-Cry3Bb) to axenic and nonaxenic larvae, we found that nonaxenic larvae were killed significantly faster than axenic larvae (Fig. 1). Compared with control larvae fed on wild-type poplar plants, we revealed that feeding of *Pa*-Cry3Bb resulted in the significant alternation of the composition and abundance of the gut microbiota (Fig. 3). It has been demonstrated that the presence of the peritrophic matrix may regulate the growth of gut microbiota (38). Aside from protecting the midgut epithelium from pathogen infection, the peritrophic matrix may also play a role in limiting immune activation in the gut (39 to 41). Feeding of *Pa*-Cry3Bb to *P. versicolora* larvae may cause peritrophic matrix damage and direct contact of intestinal bacteria with epithelial cells. Our data further demonstrated that gut bacteria could overcome the intestinal barrier and reach the hemocoel in nonaxenic larvae (Fig. 2), causing septicemia and thus accelerating the mortality of *P. versicolora*. This is in agreement with previous reports that *Bt* treatment could result in the translocation of some insect gut bacteria from the midgut to the hemocoel, and become from commensal bacteria to pathogens. However, since no gut bacteria are present in axenic larvae, the contribution to the killing process by gut microbiota was abolished. The death of axenic larvae is probably only due to the toxicity of administered Cry3Bb. After being reintroduced into axenic larvae with individual species of intestinal bacteria, the bacterium-inoculated larvae were killed faster than axenic larvae, at a similar rate to nonaxenic larvae (Fig. 4). The results clearly indicated that gut microbiota accelerated the Cry3Bb-induced mortality of *P. versicolora*.

Although most studies of the *Bt* insecticidal mechanism were carried out in Lepidopteran insects, the role of the gut microbiota in *Bt* pathogenicity in Coleopteran insects received less attention. The gut bacteria and immune system have been reported to play important roles in larval susceptibility to *Bt* in *Lymantria dispar* and Spodoptera littoralis (23, 25, 42). Suppression of insect immunocompetence was proposed as a strategy to enhance the *Bt* insecticidal activity in *S. littoralis* (42). The immune response of P. versicolora involved in the Cry3Bb insecticidal mechanism will need further investigation.

**FIG 5:**
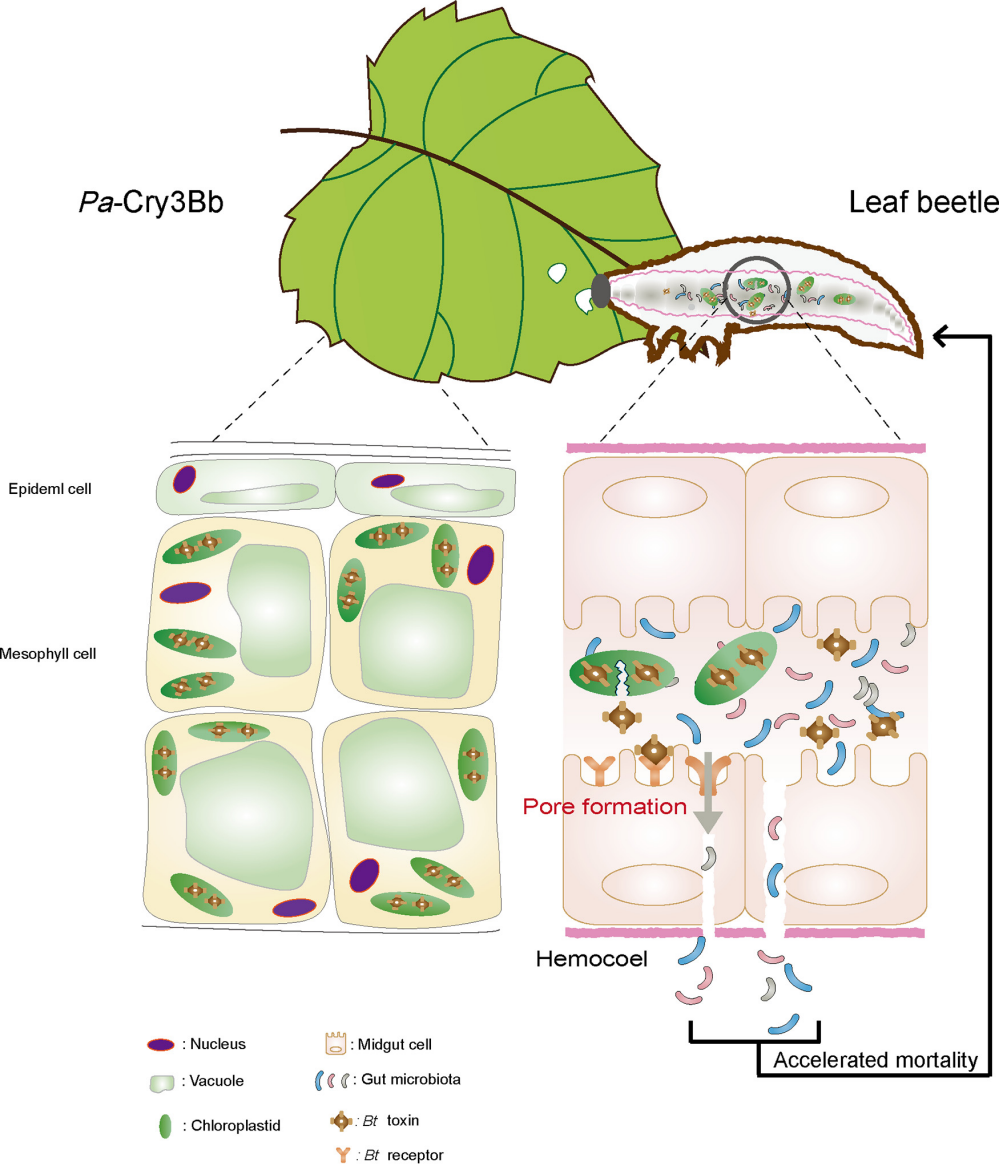
Schematic diagram of *Bt* crystal protein Cry3Bb-mediated insect killing. In the gut of the insect, Cry3Bb can be taken up by midgut epithelial cells and loaded onto the *Bt* protein machinery. *Bt* toxin binding to the receptor, and membrane insertion to form a pore. Gut bacteria can translocate from the gut lumen into the hemocoel, thus resulting in accelerated mortality of their host insect (23).

In conclusion, our study demonstrates the important role of gut microbiota in the acceleration of Cry3Bb-mediated killing of *P. versicolora* larvae (Fig. 5). These findings provide new insight into the complex interplay between transplastomic *Cry3Bb*-expressing plants and the insect pest. Moreover, these results have potential from an applied perspective, setting the stage for improving the efficiency of plastid transformation technology for pest control by expression of *Bt* toxins.

## MATERIALS AND METHODS

### Preparation of *P. versicolora* and obtaining axenic larvae.

*P*. *versicolora* adults were collected from willows at Sha Lake Park in Wuhan (30.35° N, 114.33 E), Hubei, China. Insects were regularly reared by feeding with detached fresh willow leaves at 28°C and 60% ± 5% relative humidity under a 16-h light/8-h dark photoperiod. Axenic larvae were prepared as described previously (22). Briefly, newly laid eggs were soaked in 40% NaOH for 1 min and subsequently in 70% ethanol for 5 min for sterilization. After washing with sterilized water and air drying, individual eggs were transferred onto Luria-Bertani (LB) solid medium. Successful removal of gut bacteria was verified by no colony-forming and no PCR amplification signal using conserved primers (338F: ACTCCTACGGGAGGCAGCA; 806R: GGACTACHVGGGTWTCTAAT) for the *16S rRNA* gene of gut bacteria (Fig. S1). The newly hatched larvae were then transferred to aseptic detached poplar leaves under sterilization conditions (28°C, 60% ± 5% relative humidity, 16 h day length). Poplar plantlets were raised aseptically on Murashige and Skoog (MS) medium containing 0.1 mg/L α-Naphthalene acetic acid (NAA) and 3% (wt/vol) sucrose in Magenta boxes. Nonaxenic larvae fed with aseptic poplar leaves were used for comparison in the next experiments.

### Bioassays.

*P. versicolora* axenic and nonaxenic larvae at second-, third-, or fourth-instar stage (*n *= 40) were used to test the insecticidal effects of being fed leaves of *Pa*-Cry3Bb transplastomic poplar plants (34). Leaves of wild-type poplar plants were used as control. During whole feeding assays, fresh poplar leaves were provided to the insects and exchanged daily. The mortality was recorded daily, and dead larvae were discarded immediately.

### Histological analysis.

The entire larval bodies (*n *= 5) were promptly fixed in 10% neutral buffered formalin with 2% dimethyl sulfoxide supplemented for 24 h and dehydrated in a series of alcohol baths (starting at 50% and progressing to 100%). After cleaning with xylol for 4 h, the sample was embedded in paraffin. Then, hematoxylin and eosin were used to create cross sections with a microtome (LEICA RM 2016, Leica Microsystems, USA). At least 10 paraffin sections in each group were examined under a fluorescence microscope (Nikon Eclipse E-200 model, Tokyo, Japan).

### Analysis of gut microbiota.

We utilized *Pa*-Cry3Bb leaves to feed the third-instar *P. versicolora* nonaxenic larvae, and leaves of wild-type axenic poplar plants at a similar stage were used as control. After *P. versicolora* were allowed to feed for 24 h, the midguts of insects were placed on ice (freezing anesthesia), disinfected in 75% ethanol for 90 s, washed in sterile water several times (43, 44), and then dissected by using a microscope under sterile conditions. Ten guts were pooled and collected in a 1.5-mL centrifuge tube. A total of four tubes were made and served as four replicates, cryopreserved at −80°C for subsequent DNA extraction. The midgut bacterial genomic DNA was extracted from collected samples using the Biomarker Blood/Cell/Tissue DNA kit (Biomarker, China) following the instructions of the manufacturer. The hypervariable region of the *16S rRNA* gene (V3 and V4 region) from genomic DNA was amplified and sequenced using an Illumina NovaSeq platform. Raw sequences were split according to their unique barcodes and trimmed of the adaptors and primer sequences using QIIME (45). Paired-end reads were merged using FLASH (version 1.2.11, http://ccb.jhu.edu/software/FLASH/). High-quality data (clean reads) were analyzed using fastp (version 0.20.0) software. In order to identify chimera sequences, the Clean Tags were compared to the reference database (the Silva database, available at https://www.arbsilva.de/), and the chimera sequences were then eliminated to get the Effective Tags. DADA2 (46) in the QIIME2 software (version QIIME2-202006) was used to denoise and produce each deduplicated sequence, which are called ASVs (Amplicon Sequence Variants). Sequences with an abundance of fewer than 5 (47) were also filtered out to obtain the final ASVs. Microbial diversity and community composition were analyzed using vegan packages in R (version 3.5.3). According to the results of species annotation, the top 10 species with the largest abundance at the genus level were selected for each sample or group, and the column sum plot of relative abundance was generated to visually view the species with a high relative abundance and their proportion at different taxonomic levels of each sample. Nonmetric multidimensional scaling (NMDS) based on weighted UniFrac dissimilarities was used to identify differences between microbial communities, and principal coordinate analysis (PCoA) based on Bray-Curtis dissimilarities were used to identify differences between microbial communities. Compositional differences in NMDS were tested using Analysis of Similarities (ANOSIM) with 1000 permutations. A Permutational Multivariate Analysis of Variance based on the weighted UniFrac distance (PERMANOVA, ADONIS) was used to test for differences in PCoA between treatments. The sequencing data were deposited at the NCBI Sequence Read Archive under BioProject PRJNA903779.

### Reintroduction of bacteria into insect guts.

Bacteria (P. putida) were previously isolated from the gut microbiota of *P. versicolora*, and their reintroduction into insect guts was performed as described (22). Briefly, bacterial cells were collected by centrifugation of an overnight culture at 4,000 rpm for 5 min. After washing with sterile PBS several times, cell pellets were resuspended in PBS and diluted to a final concentration of approximately 1 × 10^6^ cells/mL (22). The bacterial suspension was then painted onto aseptic poplar leaves (approximately 1 × 10^4^ cells/cm^2^) and fed to third instar axenic *P. versicolora* larvae (*n *= 40). The bacteria-coated poplar leaves were replaced every day. Survival was recorded daily.

### Statistical Analysis.

Survival curves were analyzed using the Kaplan-Meier method, and the log-rank test was used to determine the significance of differences between two groups. A *P* value of <0.05 was considered significantly different. Data were analyzed using SPSS version 21.0 (IBM). Unpaired comparisons between two groups were analyzed using a Student's *t* test.
