# Supplementary material for: Gut Microbiota Accelerate the Insecticidal Activity of Plastid-Expressed Bacillus thuringiensis Cry3Bb to a Leaf Beetle, Plagiodera versicolora
Source: Microbiol Spectr. 2023 Mar 28;11(2):e05049-22. doi: 10.1128/spectrum.05049-22 (PMC10101071; doi:10.1128/spectrum.05049-22)
Supplement: Supplemental file 1 — Supplemental material. Download spectrum.05049-22-s0001.pdf, PDF file, 0.1 MB [file spectrum.05049-22-s0001.pdf]

Supporting information

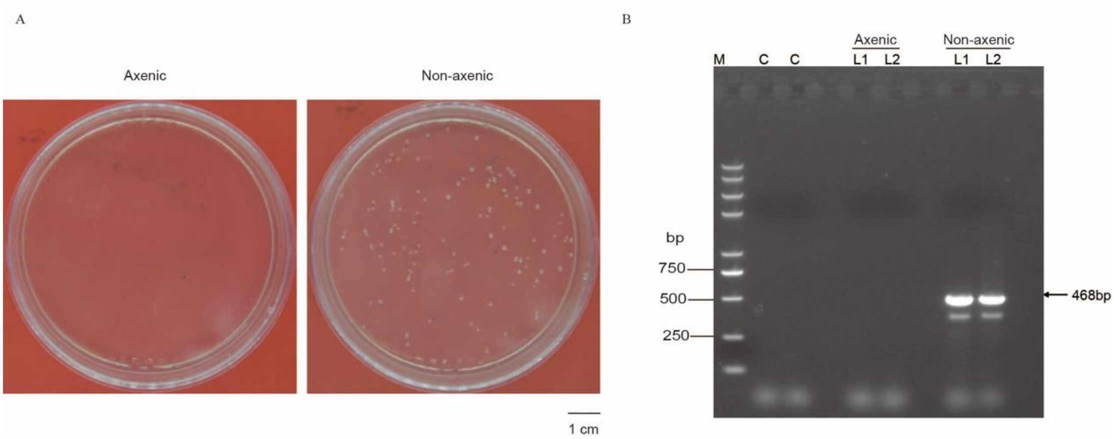

Figure S1 Verification of the axenic status of *P. versicolora* larvae derived from surface-sterilized eggs. The absence of bacteria from the axenically reared insects was confirmed by (**A**) the lack of bacterial colonies forming on LB agar plates (n = 10, gut extracts were diluted 10^4 fold with sterilized water), and (**B**) the absence of PCR amplicons from reactions using universal *16S rRNA* gene primers for bacteria (n = 10). L1 and L2 are the individual larva no. 1 and 2, M: DNA size marker. C: negative control (no template DNA added).

S1 Table. Comparison of diversity indices (mean ± SEM, *n* = 5) of the *P. versicolora* gut bacterial community in larvae fed with leaves of *Pa*-wt and *Pa*-Cry3Bb.

**Sample**	**Chao1 index**	**Observed_otus index**	**Shannon index**	**Simpson index**
CK-1	195.500	195	3.157	0.772
CK-2	145.200	145	2.738	0.787
CK-3	328.200	328	3.937	0.850
CK-4	234.100	234	3.040	0.772
Cry3Bb-1	180.750	180	4.060	0.891
Cry3Bb-2	400.167	400	4.588	0.883
Cry3Bb-3	393.000	392	4.768	0.913
Cry3Bb-4	158.200	158	4.343	0.924

ASV richness and diversity values were calculated for genus-level ASVs.

Data were analyzed using Student’s t test.

Chao1 index: The total number of species included in the community samples was estimated.

Observed_otus index: The number of species observed visually.

Shannon index: the total number of categories in the sample and their proportion.

Simpson index: The diversity and evenness of species distribution within a community.
